# CircXPO5 Plays a Neuroprotective Function in the Lateral Geniculate Nucleus of Glaucoma by Regulating GRIN2A

**DOI:** 10.3390/brainsci12060780

**Published:** 2022-06-14

**Authors:** Zhichao Yan, Mingying Lai, Yu Jia, Caibin Deng, Yehong Zhuo

**Affiliations:** 1 Department of Glaucoma and Neuro-Ophthalmology, Shenzhen Eye Hospital, Shenzhen Eye Institute, Jinan University, School of Optometry, Shenzhen University, Shenzhen 518040, China; laimydoc@163.com; 2 Department of Ophthalmology, The Second Affiliated Hospital of Guangzhou Medical University, Guangzhou 510260, China; 3 State Key Laboratory of Ophthalmology, Zhongshan Ophthalmic Center, Sun Yat-sen University, Guangdong Provincial Key Laboratory of Ophthalmology and Visual Science, Guangzhou 510060, China; yuyu.jia@outlook.com (Y.J.); dengcb@mail.sysu.edu.cn (C.D.)

**Keywords:** circXPO5, miR-330-5p, GRIN2A, glaucoma

## Abstract

Purpose: Previous studies have found the neurodegeneration and atrophy of glaucomatous lateral geniculate nucleus (LGN), but the mechanism is still unknown. Circular RNA (circRNA) plays some important roles in physiological and pathological progression of the disease. In this study, we focused on the differentially expressed circRNAs and the mechanism for circXPO5 in LGN degeneration in a macaque glaucoma model. Methods: Using RNA-seq, we analyzed the differentially expressed circRNAs in a macaque glaucoma model. An RT-QPCR was used to check the expression of selected differentially expressed circRNAs, candidate miRNAs and mRNAs. A competing endogenous RNA (ceRNA) network analysis was performed to examine the mechanism of circXPO5 action. Results: circXPO5 significantly decreased in the glaucoma model and a ceRNA network analysis revealed that circXPO5 can bind to miR-330-5p, which also binds to GRIN2A (ionotropic receptor NMDA type subunit 2A). QPCR detection showed a decrease in GRIN2A and an increase in miR-330-5p. Conclusions: Our earlier studies revealed that the GRIN2A gene regulates the calcium signal pathway. Decreasing of GRIN2A related with neuron apoptosis and neurodegeneration. These findings indicate that the reduction in circXPO5 may have a protective effect on neuronal apoptosis in the visual central system of glaucoma.

## 1. Introduction

Glaucoma is the leading reason for irreversible blindness all over the world [1]. There were 64.3 million people affected by glaucoma worldwide in 2013, and this number is predicted to grow up to 76.0 million in 2020 and 112 million in 2040 [2]. The common feature of glaucoma is the loss of the retinal ganglion cells (RGC) and cupping of the optic disc [1,3]. Besides the high incidence of glaucoma, especially in older people, the pathogenesis and mechanism of glaucoma is still poorly understood [4]. Increasing intraocular pressure (IOP) is the most common pathological factor for glaucoma [1]. Reducing intraocular pressure is the only approach for intervention and treatment of glaucoma so far, including drugs, lasers and surgery [1]. However, not all glaucoma patients benefit from these approaches. Even if the IOP of some patients is well controlled, their visual function might still be further impaired [5]. It is plausible that there are other factors and/or mechanisms that contribute to glaucoma progression and vision loss, warranting further investigation.

The visual center is mainly composed of the lateral geniculate nucleus (LGN) and the visual cortex. In humans, the transmission of visual information is processed by a series of neurons in the retina and transmitted to the LGN (in rodents, to the superior colliculus) and then to the visual cortex. Any obstacles in the visual circuit will affect the transmission of visual signals. In glaucoma, with increased IOP leading to damage of the RGC, the effect may diffuse to the LGN and the visual cortex, leading to pathological changes in the LGN [6,7]. In recent years, several studies reported that the LGN of primate glaucoma animal models and glaucoma patients have neurodegeneration and atrophy [7,8]. Our previous research results also revealed that there are neuronal apoptosis and Alzheimer’s disease (AD)-like degenerative changes in the visual center of chronic glaucoma rhesus monkeys [6]. These studies indicate that glaucoma is not simply an RGC-injury disease, but a complex neurological disease, suggesting that the repair or transplantation of functional RGC may not effectively restore vision. Better understanding of the mechanisms that are involved in the damage and protection of the visual center of glaucoma is urgently needed.

The glutamate receptor channels mediate most of the fast excitatory synaptic transmission in the mammalian central nervous system. One of the subtypes of glutamate receptors are N-methyl-D-aspartate receptors (NMDAR) [9]. NMDAR is highly permeable to Ca^2+^ and is thought to play an important role in synapse formation and synaptic plasticity, mediating learning and memory functions [10]. On the other hand, increased NMDAR activity results in excitotoxicity and has been implicated in many neurodegenerative disorders, such as Parkinson’s, Alzheimer’s and Huntington’s disease [11,12]. Functional NMDAR is a heterotetramer that is composed of two GluN1 and two GluN2 subunits [13]. The GluN2A subunit is encoded by the GRIN2A gene [14]. In an integrated bioinformatics analysis of AD, GRIN2A is a hub gene for the miRNA interaction network, and its expression is decreased in AD samples [15], suggesting that GRIN2A plays some roles in calcium signaling and neurodegeneration in Alzheimer’s disease. Aβ promotes the hyperphosphorylation of intracellular Tau protein, interferes with mitochondrial function, causes disturbance of intracellular calcium, and finally induces apoptosis [16,17]. Our previous research results also found that Aβ is deposited in the atrophic visual center [6]. Whether Aβ participates in the ER stress-mediated glaucoma visual central neuron apoptosis pathway is unclear.

Circular RNA (circRNA) is a type of non-coding RNA (ncRNA) that was only discovered in recent years [18]. Abnormally expressed circRNAs play some important roles in the pathological processes of human diseases [19]. Their mechanism of action includes competitive endogenous RNA (ceRNA) for miRNAs, binding with proteins, as well as directly translation for protein. Among these mechanisms, ceRNA is the most frequently reported. It has been confirmed that circRNA-miRNA is related to protein-coding genes and genetic biological processes [20]. circRNAs, especially, as a competitive endogenous RNA (ceRNA), usually regulate target genes through competitive binding with miRNA, and acts as a sponge of miRNA, thereby releasing miRNA’s inhibitory effect on its target genes.

In this study, we aimed to explore the functional role of circRNAs in LGN degeneration in a macaque glaucoma model. Using high-throughput sequencing and a co-expression network analysis, we found that the expression of circXPO5 is correlated to GRIN2A mRNA. Further analysis showed that circXPO5 and GRIN2A can compete with miR-330-5p as ceRNA. Our results indicate that the decrease in circXPO5 may play a protective role in apoptosis of the central optic neurons in glaucoma.

## 2. Materials and Methods

### 2.1. Macaque Glaucoma Model and LGN Samples Preparation

Tissue samples of a macaque (*Rhesus macaque*) glaucoma model were obtained from our earlier studies [6]. This process strictly adhered to the ARVO Statement for the Use of Animals in Ophthalmic and Vision Research, and was approved and monitored by the Institutional Animal Care and Use Committee of Zhongshan Ophthalmic Center (Permit Number: SYXK (YUE) 2010-0058). The method for the macaque glaucoma model was generated as previously described [6]. In summary, bilateral chronic IOP elevation was induced in 3 rhesus monkeys by laser photocoagulation of the trabecular meshwork in both eyes. Another 3 normal monkeys that did not receive laser photocoagulation were used as controls. IOP was monitored weekly between 9:00 a.m. and 12:00 p.m. with a Tono-Pen XL tonometer before and after laser treatment. If the IOP was not consistently higher than 24 mm Hg, additional laser treatments were performed at least 2 weeks after the previous treatment until stable ocular hypertension was achieved. After identification and confirmation of the successful construction of the glaucoma model, all 6 model animals were euthanized, and their LGN quickly dissected as described [6]. The separated samples were quickly frozen in liquid nitrogen and transferred to −80 °C freezer.

### 2.2. RNA Extraction and High Throughput RNA Sequencing and Data Analysis

RNA that was extracted by a Trizol reagent (Invitrogen) with RIN > 6.0 was utilized to construct an rRNA depletion library (NEBNext^®^ Ultra^TM^ Directional RNA Library Prep Kit) according to the manufacturer’s instructions. Whole transcriptome sequencing data sequenced by a HiseqTM Sequencer were filtered (removing the adaptor sequences, reads with >5% ambiguous bases (noted as N) and low-quality reads containing more than 20 percent of bases with qualities of <20) and mapped to monkey genome (utilizing HISAT2 [21]. HTSeq was used to calculate the gene count of mRNA and lncRNA [22].

### 2.3. CircRNA Prediction and Data Analysis

The pipeline ‘acfs’, which was publicly available at https://code.google.com/p/acfs/ (accessed on 2 August 2014), was used to identify the circRNA in each sample, including the following steps:

Unmapped reads collection: BOWTIE2 version 2.2.5 was used as the mapping method to the respective reference genome [GRCH37.p13 NCBI].

CircRNA identification: Unmapped reads were collected to identify the circRNA utilizing BWA mem (bwa mem −t 1 −k 16 −T 20): partial alignments of segments within a single read that mapped to (a) regions on the same chromosome and no more than 1 Mb away from each other; (b) on the same strand; (c) but in reverse order were retained as candidates supporting head-to-tail junction. The strength of potential splicing sites supported by these candidate head-to-tail junction reads was then estimated using MaxEntScan33. The exact junction site was determined by selecting the donor and acceptor sites with the highest splicing strength score. Candidate circRNA were reported if the head-to-tail junction was supported by at least two reads and the splicing score was ≥10.

Expression analysis: To estimate the expression of circRNA, we realigned all the unmapped reads to the circRNA candidates using the BWA mem under the following parameter (bwa mem −t 1 −k 16 −T 20). As for most of the circRNA there is no direct evidence for their exact sequence, so we filled in the sequence using an existing exon annotation. The sequence at the 5′ end was concatenated to the 3′ end to form circular junctions. Reads that mapped to the junction (with an overhang of at least 6 nt) were counted for each candidate.

### 2.4. DEG Analysis and ceRNA Relation Prediction

A differentially expressed genes (including mRNA, lncRNA and circRNA) analysis was applied utilizing DESeq [23] under the following criteria: Fold Change > 2; FDR < 0.05. lncRNAs; circRNAs; and mRNAs with expression levels that shared meaningful correlations were subjected to a ceRNA analysis. We searched for potential miRNA response elements (MREs) among the lncRNA, circRNA, and mRNA sequences, and overlaps between the same miRNA seed sequence binding sites in both lncRNA/circRNA and mRNA sequences were considered to be predictive of lncRNA/circRNA-miRNA-mRNA interactions. miRNA binding sites were predicted using miRbase (http://www.mircode.org/ (accessed on 1 January 2021)), and miRNA–mRNA/miRNA–lncRNA/miRNA–circRNA interactions were predicted using a miRanda package [24].

### 2.5. Co-Expression Analysis

We present gene CoExpNets to find the interactions among genes [25]. Gene CoExpNets were built according to the normalized signal intensity of specific expression genes. For each pair of genes, we calculate the Pearson correlation and choose the significant correlation pairs to construct the network. Within the network analysis, degree centrality is the simplest and most important measure of the centrality of a gene within a network that determines the relative importance. Degree centrality is defined as the link numbers one node has to the other. Moreover, to investigate some properties of the networks, W-cores in graph theory were introduced as a method of simplifying the graph topology analysis. A W-core of a network is a subnetwork in which all the nodes are connected to at least W other genes in the subnetwork. A W-core of a protein-protein interaction network usually contains cohesive groups of proteins. The purpose of a network structure analysis is to locate the core regulatory factors. In one network, the core regulatory factors connect most adjacent genes and have the biggest degrees. While considering different networks, the core regulatory factors were determined by the degree differences between two class samples. They always own the biggest degree differences.

### 2.6. Functional Analysis

#### 2.6.1. GO Analysis

A gene ontology analysis was performed to facilitate elucidating the biological implications of differentially expressed mRNA. We downloaded the GO annotations from NCBI (http://www.ncbi.nlm.nih.gov/ (accessed on 29 May 2022)); UniProt (http://www.uniprot.org/ (accessed on 29 May 2022)); and the GO (http://www.geneontology.org/ (accessed on 29 May 2022)). The genes that were annotated by an integrated GO database were set up as the background genes and based on the background information; Fisher’s exact test was applied for the GO analysis with significant *p*-value calculated, and FDR was used to correct the *p*-values.

#### 2.6.2. Pathway Analysis

A pathway analysis was used to find out the significant pathway of the differential genes according to the KEGG database. We used the Fisher’s exact test to select the significant pathway, and the threshold of significance was defined by FDR < 0.05.

### 2.7. miRNA Binding Prediction

We utilized the miRanda and RNAhybrid as the tools for predicting differentially expressed miRNA target on mRNA.

## 2.8. Fluorescence Quantitative PCR (QPCR)

The SYBR^®^ Green QPCR mix (Thermo) was used for the qRT-PCR analysis. Reaction was performed on a Roche LightCycler^®^ 480II PCR instrument (Basel, Switzerland). GAPDH was used as an internal standard control. The relative RNA expression levels were calculated by the 2^−ΔΔCT^ method. The primers that were used in the study are listed in the Supplementary Materials.

## 2.9. Statistics

All statistical analyses were performed by SPSS 20.0 (SPSS, Chicago, IL, USA), R software (version 3.6.1), and GraphPad Prism 8.0 (GraphPad Software Inc., San Diego, CA, USA). A student’s *t*-test was used to analyze the differences between the groups. The correlations between the groups were assessed using a Pearson correlation analysis. *p* values less than 0.05 were considered statistically significant.

## 3. Results

### 3.1. GRIN2A Is Decreased in LGN of Macaque Glaucoma Model

**Figure 1:**
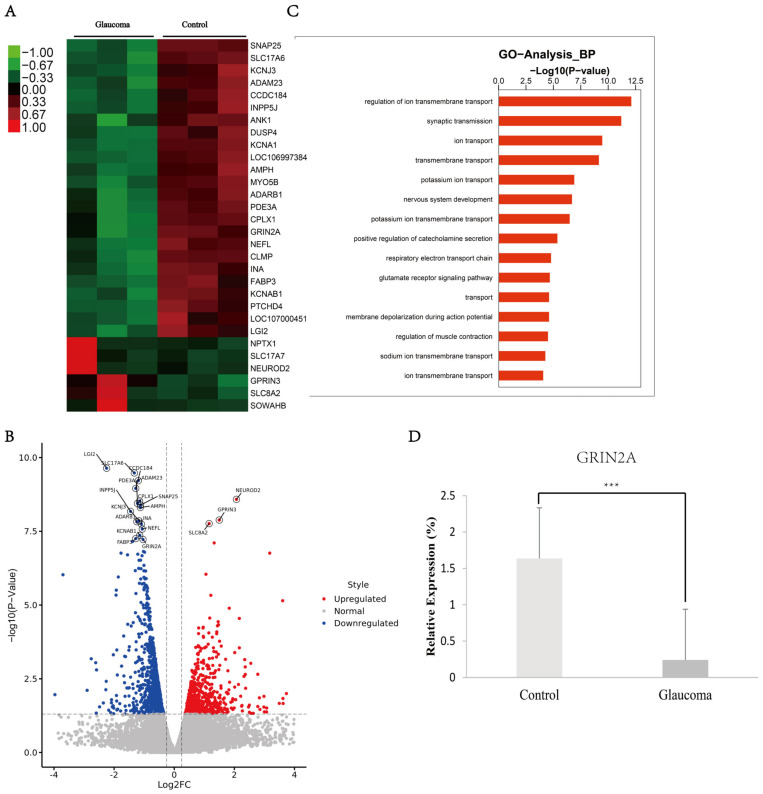
Differentially expressed genes in LGN of macaque glaucoma model. (**A**) Differentially expressed genes are calculated, heat-map showed the top differential genes; (**B**) volcano map of selected differentially expressed genes; (**C**) GO pathway analysis for differentially expressed genes; (**D**) QPCR detection of GRIN2A in LGN of macaque glaucoma model. ***, *p* < 0.05.

The progressive neurodegeneration of the LGN is a key event in the blinding process of glaucoma. However, little is known about the gene expression during the neurodegeneration of the LGN. In order to verify the changes in the transcriptome of the LGN tissues in the macaque glaucoma model, we collected the LGN tissues of three glaucoma and three control macaque monkeys, extracted the total RNA and performed a high throughput RNA-seq analysis. After the GO pathway analysis, the “Ion channel activity” pathway would possibly be one of the most changed in macaque glaucoma models, which includes GRIN2A (Figure 1A–C). A reported decrease in GRIN2A plays some roles in calcium signaling and neuro-degradation in Alzheimer’s disease [15]. The results revealed that GRIN2A was more greatly decreased in the macaque glaucoma models than in the control samples (Figure 1D).

### 3.2. Differential Expression of circRNA in LGN of Macaque Glaucoma Model

**Figure 2:**
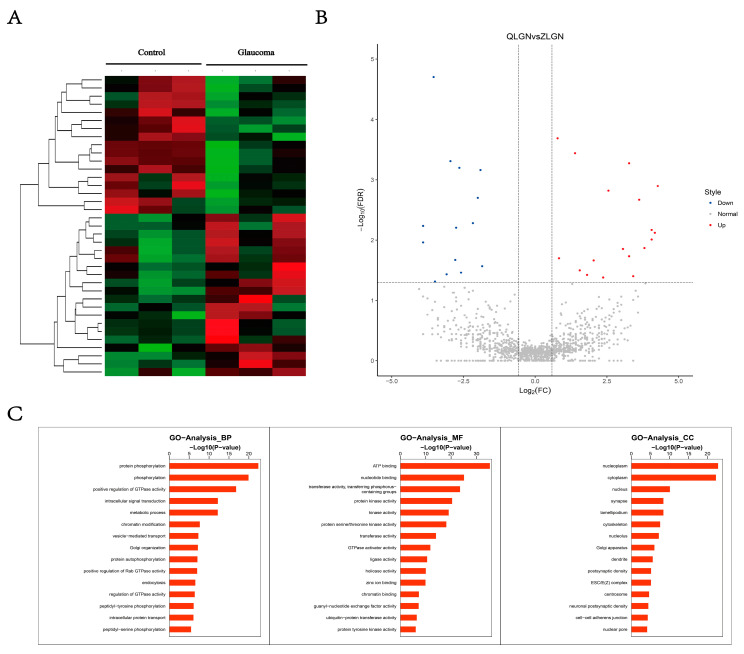
Differentially expressed circRNAs in LGN of macaque glaucoma model. (**A**) Heat map of differentially expressed circRNAs; (**B**) volcano map of differentially expressed circRNAs; (**C**) GO pathway analysis for differentially expressed circRNA host genes.

In order to ensure which circRNA work as the miRNA sponge to regulate the expression of GRIN2A, we analyzed the circRNAs in transcriptome data. We identified 37 differentially expressed circRNAs, 20 of them were up-regulated in macaque glaucoma model LGN samples, compared to the control group (Figure 2A,B). All these differentially expressed circRNAs are listed in Supplementary Table S1. The GO pathways of different circRNA host genes were also analyzed: “Protein phosphorylation” and “ATP binding” were the most changed pathways (Figure 2C). The expression pattern of related gene changes during the pathological process may lead to the transformation of the circRNA-miRNA network into a state that is favorable for the pathological process.

### 3.3. Co-Expression Network Analysis

**Figure 3:**
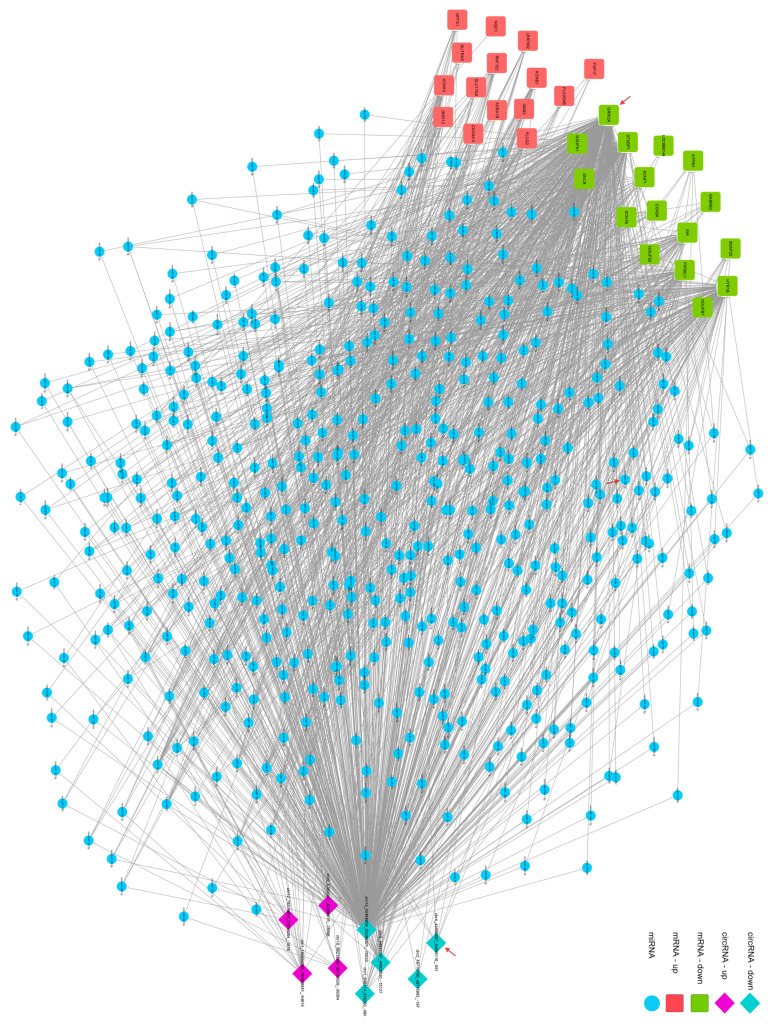
Interaction network analysis for differentially expressed genes (arrows are target genes).

**Figure 4:**
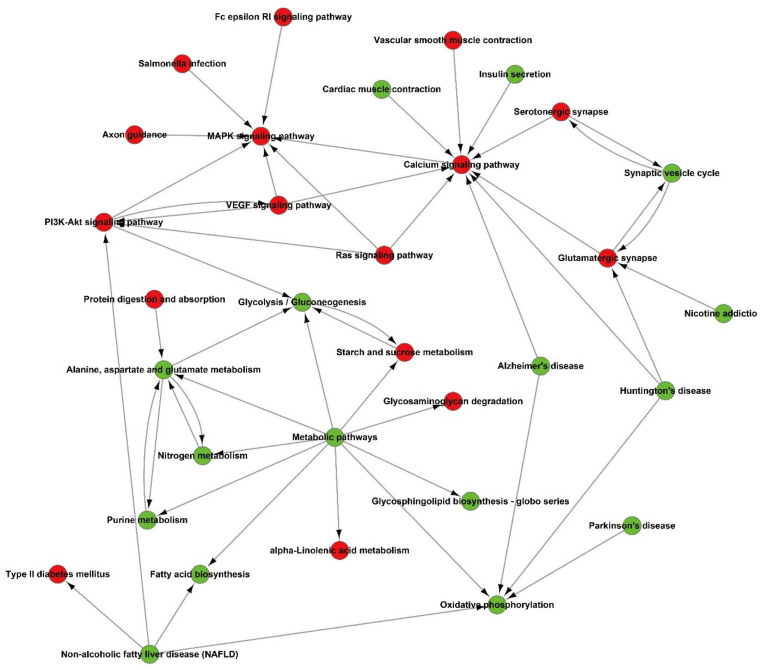
Interact network analysis for differentially expressed genes and pathways.

A ceRNA network analysis is widely used to find lncRNA-miRNA-mRNA regulatory units [15,26,27,28]. circRNA as a type of non-coding RNA also works as a miRNA sponge to competitive combined miRNAs. Changes in the circRNA-miRNA-mRNA network in the pathological process can be calculated by a co-expressing analysis. Co-expression refers to the high degree of similarity between the expression patterns of two genes in a set of samples. Gene co-expression relationships that are extracted from gene sequencing data can provide clues for the functional annotation of non-homologous genes, as well as new ideas for protein co-expression and interaction research. We performed a gene co-expression analysis (Figure 3 and Figure 4) and identified four candidate circRNAs whose expression is correlated with GRIN2A: chr 1_ 848114_838250_-9864; chr 2_ 48776160_48775963_-197; chr 4_ 44595667_44595116_-551; and chr 8_ 44017157_44002030_-15127.

### 3.4. miRNA Prediction between Candidate circRNAs and GRIN2A

**Figure 5:**
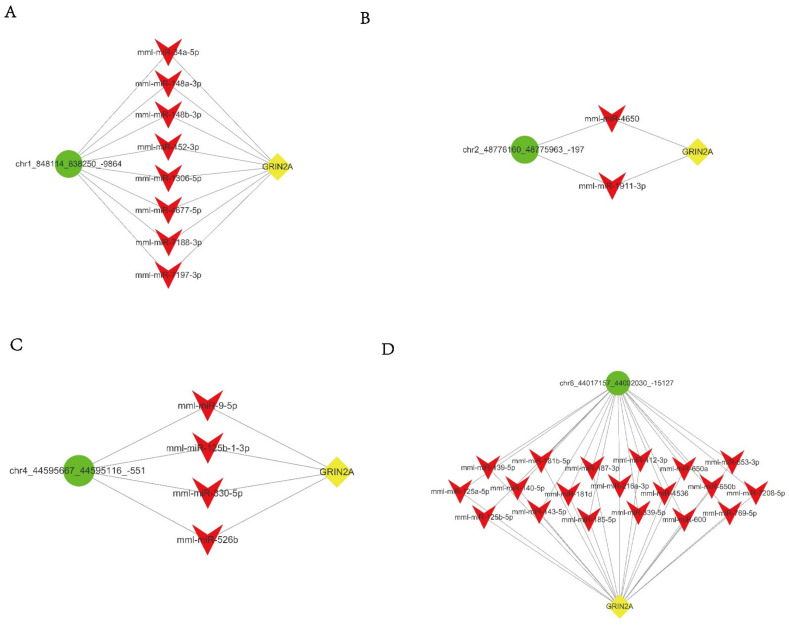
Prediction of miRNAs between candidate circRNAs and GRIN2A. (**A**–**D**) Predicted miRNAs for 4 candidate circRNAs.

CircRNAs can regulate the expression of downstream genes through the competitive binding of miRNAs. As shown in Figure 5, miRNAs for the four candidate circRNAs and GRIN2A were predicted by miRanda and RNAhybrid tools.

### 3.5. CircXPO5 Regulates GRIN2A through Sponging miR-330-5p

**Figure 6:**
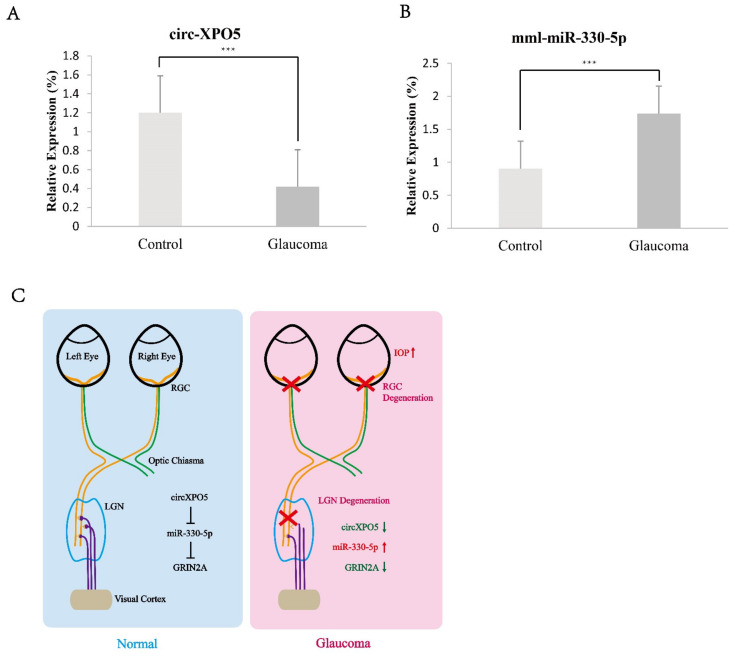
Detection expression of circXPO5 and miR-330-5p in LGN of macaque glaucoma model. (**A**) QPCR detection of circXPO5; (**B**) QPCR detection of miR-330-5p; (**C**) mechanism model of circXPO5/miR-330-5p/GRIN2A in glaucoma LGN degeneration. ***, *p* < 0.05.

Through QPCR detection, we examined the expression of four candidate circRNAs. Only chr 4_ 44595667_44595116_-551 corresponded to the expression change of GRIN2A in the macaque glaucoma LGN samples (Figure 6A). chr 4_ 44595667_44595116_-551 is generated from the XPO5 gene—we name it circXPO5 hereafter. We checked the expression of miRNAs which correspond to circXPO5 and found that miR-330-5p were upregulated in the macaque glaucoma LGN samples (Figure 6B). As a result of LGN degeneration, circXPO5 is downregulated, leading to the release of miR-330-5p and consequently decreasing GRIN2A expression (Figure 6C).

## 4. Discussion

With the increase in IOP, the interruption of the retrograde transmission of neurotrophic factors in the optic nerve axon of the optic nerve head is considered the main mechanism of RGC death [29], highlighting the need to protect RGCs from damage [30,31], or replace them with stem cell-derived counterparts [32,33]. In recent years, studies have found degeneration and atrophy of the visual center of glaucoma [8,33]. For example, changes in the eye dominance column of the visual cortex and its cellular metabolic activity have been observed [34,35,36]. Recent technological advances have allowed detection of atrophy of the visual center at a morphological as well as a functional level [37,38]. Recent studies have found that the degeneration of LGN may be related to endoplasmic reticulum stress [8] and peroxynitrate-mediated oxidative stress damage [39], but the specific damage signaling pathway is still unclear. In recent years, many scholars believe that the pathogenesis of POAG may be related to a variety of system diseases, such as AD and Parkinson [40,41]. AD is one of the diseases related to glaucoma which has attracted more attention [40]. Recently, it has been reported that amyloid β (Aβ) is related to the occurrence of RGC apoptosis in glaucoma [42,43], and studies have also shown that the increase in intraocular pressure promotes the expression of Aβ and apoptosis of RGCs [44]. Similarly, Tau (another characteristic marker of AD) was found to increase in the plasma of glaucoma patients, and studies on mouse models of glaucoma also confirmed that phosphorylated Tau (p-Tau) protein is related to retinal cell death [45,46]. Our previous research results also found that there are neuronal apoptosis and Alzheimer’s disease-like degenerative changes in the visual center of rhesus monkeys with chronic glaucoma [6]. The molecular mechanism and related regulatory mechanisms are still unknown.

Endoplasmic reticulum (ER) is the most important calcium storage area of the cell. There are many channels for pumping calcium ions on the endoplasmic reticulum membrane, such as SERCA (troponin/endoplasmic reticulum Ca^2+^ ATPase) and Na^+/^Ca^2+^ exchangers, which can pump calcium ions from the cytoplasm into ER to counter the concentration gradient. The channels that release calcium ions on the endoplasmic reticulum membrane mainly include inositol 1,4,5 triphosphate receptor (IP3R) and ryanodine receptor (RyR). In order to balance the calcium ions inside and outside the cell, it is performed through voltage-gated channels, mechanically gated channels and N-methyl-D-aspartate receptors (NMDAR), and other ligand-gated channels on the cell membrane. NMDAR is a subtype of ionotropic glutamate receptors which plays an important physiological role in the development of the nervous system. After the NMDA receptor is activated, it is mainly permeable to Ca^2+^ and mediates a continuous and slow depolarization process. Endoplasmic reticulum stress (ER stress) is caused by the imbalance of Ca^2+^ homeostasis, which is also one of the important causes of cell apoptosis [47]. Studies have confirmed that by activating caspase-12 in the ER, the cytotoxicity of Aβ is related to ER stress, thereby activating the caspase family apoptotic pathway in mitochondria [48,49]. The study also found that in the process of Aβ deposition causing AD hippocampal neuron damage, extracellular Aβ activates cell surface receptors (such as NMDAR, GPCR, Fas/TNFR, etc.) to stimulate cell apoptosis. Furthermore, hyperphosphorylation of intracellular Tau protein interferes with mitochondrial function, causes disturbance of intracellular calcium, and finally induces apoptosis [16,17]. Another pathway of Aβ toxicity is apoptosis that is mediated by the death receptor caspase-8 activation [50].

Here, our results of high-throughput sequencing analysis of the glaucoma model LGN show that the abnormal expression of AD-related genes participates in AD-related pathways, and ultimately participates in calcium signaling pathways. The calcium ion signaling pathway plays an important role in cell apoptosis that is mediated by endoplasmic reticulum stress. Previous studies have found that the degeneration of the LGN visual center in an animal model of glaucoma is related to endoplasmic reticulum stress [6]. In previous studies on this subject, it was also found that the expression of the main ER stress protein GRP94 and the downstream signal-caspase 9 were elevated [51]. Our previous research results also found the deposition of Aβ in the atrophic visual center [6], so it is still unclear whether Aβ is involved in the ER stress-mediated apoptosis pathway of glaucoma visual central neurons. In this work, the results of screening LGN pathogenic genes found that core mRNA-GRIN2A was significantly reduced. Moreover, our results suggest that circXPO5 expression is related to GRIN2A mRNA.

A ceRNA network analysis can quickly and effectively find molecules with regulatory relationships. A ceRNA analysis was reported to find the potential biomarkers for primary open-angle glaucoma [27], and find the potential biological target molecules that are related to primary open-angle glaucoma [28]. CircRNAs usually play a role as ceRNA for miRNAs, and miRNAs play some regulatory roles on mRNA. Our results found that the expression of circXPO5 corresponds to GRIN2A mRNA. A ceRNA net analysis found that several miRNAs can link circXPO5 and GRIN2A. After the QPCR analysis, miR-330-5p was shown to be a linker for circXPO5 and GRIN2A. Our results indicate that the decrease in circXPO5 may play a protective role in the apoptosis of the central optic neurons in glaucoma. Studies reported that miR-330-5p expression is elevated in AD, which is consistent with our results. The study showed that miR-330 promotes the formation of the dendritic spines of hippocampal neurons in the early stage of AD through the Rpph1/miR-330-5p/CDC42 axis [52].

This work was carried out in non-human primates that share a high degree of homology with humans. These results provide valuable reference information for future related treatment research. However, this work was only a cross-sectional analysis study; a longitudinal intervention can be carried out in mice in the future.

## Data Availability

Data is contained within the article or Supplementary Materials.

## Supplementary Materials

The following supporting information can be downloaded at: https://www.mdpi.com/article/10.3390/brainsci12060780/s1. Table S1: Differentially expressed circRNA between glaucomatous and normal LGN samples.
